# Functional and Radiological Outcome of Unstable Intertrochanteric Fracture Post Dynamic Hip Screw Fixation

**DOI:** 10.7759/cureus.4360

**Published:** 2019-04-01

**Authors:** Muhammad T Lakho, Asif A Jatoi, Muhammad Khanzada Azfar, Aijaz Ali, Safiya Javed, Anisuddin Bhatti, Musa Karim

**Affiliations:** 1 Orthopedics, Jinnah Postgraduate Medical Center, Karachi, PAK; 2 Orthopedics, Jinnah Postgraduate Medical Center, Karachi , PAK; 3 Pathology, Isra University, Hyderabad, PAK; 4 Statistics, National Institute of Cardiovascular Diseases (NICVD), Karachi, PAK

**Keywords:** intertrochanteric fractures, radiological outcome, dynamic hip screw, functional outcome

## Abstract

Introduction

Intertrochanteric fractures are the most frequently operated fracture type and have the highest postoperative fatality rate. The most commonly used devices are the dynamic hip screw (DHS) with side plate assemblies and proximal femoral nail (PFN). The aim of this study was to determine the functional and radiological outcome of unstable intertrochanteric fracture post DHS fixation at a tertiary care hospital in Karachi, Pakistan.

Methods

A study was carried out in the department of orthopedics at the Jinnah Postgraduate Medical Center (JPMC), from 12th June 2016 to 8th September 2017. A total of 106 patients between 18 and 75 years of age with unstable intertrochanteric fracture were included. Those patients who had multiple injuries and open fractures, subtrochanteric fractures, intracapsular fractures neck of femur, pathological fractures and patients who were non-ambulatory prior to their injury were excluded. Functional outcomes were measured both during pain using the visual analog scale and range of motion on goniometer between 80 and 100 degrees. Satisfactory outcomes were measured after three months.

Results

A total of 106 patients with the radiological diagnosis of the unstable intertrochanteric fracture having post DHS fixation were included in this study. Out of which, 69.8% (74) were males and the mean age was 66.61 ± 7.79 years over the range of 50 to 80 years. Patients with type II diabetes were 22.6% (24) with a mean duration of 4.3 ± 8.37 years. At the end of three months, no pain was reported in 82.1% (87) patients and 85.8% (91) patients had normal function. The satisfactory radiological outcome was observed in 86.8% (92) patients. Overall, acceptable outcomes were observed in 81.1% (86) patients at the end of three months.

Conclusion

The treatment of unstable intertrochanteric fracture with dynamic hip screw (DHS) fixation results in better outcomes. In our study, we observed acceptable outcomes in a vast majority, 81.1%, of patients after three months of DHS fixation of the unstable intertrochanteric fracture.

## Introduction

Intertrochanteric fractures are most frequently seen in older people mainly with osteoporotic bones. These fractures frequently occur due to falling [1]. Approximately 50% of unstable fractures in elderly people constitute intertrochanteric fracture for almost half the hip fractures. Different studies have reported that hip fracture depends on the distribution of age. Almost 97% of people suffer over the age of 50 years and these incidents increased with their age [2-3]. Operative treatment is the main modality treatment of intertrochanteric fractures [2]. Almost annually, five million cases of hip fractures were estimated by the year 2050. Therefore, more research efforts appear critical to improving the outcomes of treatment modalities for hip fracture [4].

Trochanteric (per trochanteric/intertrochanteric), as well as sub-trochanteric fractures, belong to the group of proximal femoral fractures, i.e. hip fractures, an entity to which femoral neck fractures is subordinated [5]. The unstable variety is difficult to be stabilized and associated with complications with the traditional dynamic hip screw (DHS) implant, which has led the surgeons to try for the new modalities of intramedullary fixation devices [2,6].

While managing fractures, in general, it is important to have a reliable classification system. A valid fracture classification should be simple enough to provide guidelines for clinical treatment, comprehensive enough to be used in clinical outcome studies, and reasonably reliable and reproducible [7].

To classify trochanteric fractures, several classification systems have been published. Most of the classifications are based on the anatomical description of the fracture patterns observed, while others are designed to provide prognostic information on the prospect of achieving and maintaining reduction or are based on the fracture mechanism [8-9]. More than 28,0000 hip fractures occur in the United States (US) every year and these numbers are expected to double by 2050. These fractures are associated with substantial morbidity and mortality and approximately 30% of the elderly die within one year of fracture [10].

Intertrochanteric fractures are common and result in considerable mortality and morbidity which causes a great financial burden to society. At present, except for comorbidities that place patients at unacceptable risk from anesthesia, surgical procedure or both, surgical treatment of intertrochanteric hip fractures is usually reserved [11].

Unstable intertrochanteric fractures treated by proximal femoral nail anti-rotation (PFNA) had a minimally invasive approach with minimal surgical trauma and provide stable fixation allowing early mobilization with full weight bearing. Our complication rate is comparable to previous studies [12]. With regard to the inter-trochanteric femoral fractures, extramedullary plates and intramedullary nails are the two most common fixation methods. Intramedullary stabilization of unstable fractures can play an essential role in the selection of an intramedullary implant. It must be ensured that it also finds suitable support in the distal fragment [13]. However, with respect to unstable fractures, intramedullary fixations with shorter lever arm have a theoretical advantage over the extramedullary implants. To fix femoral neck fractures, two or three cannulated screws are implanted at an angle of 135 degrees, 145 degrees, and 150 degrees [14].

Despite the advancements in the clinical care of the patients, management of unstable intertrochanteric fractures is a clinical challenge for orthopedic surgeons. The aim of this study was to determine the functional and radiological outcome of unstable intertrochanteric fracture post DHS fixation at tertiary care hospital, Karachi, Pakistan.

## Materials and methods

A study was carried out at the department of orthopedics, Jinnah Postgraduate medical center (JPMC) from 12th June 2016 to 8th Sept 2017. A total of 106 patients with unstable intertrochanteric fractures ages ranging from 18 to 75 years who had the ability to walk independently with aids were allowed prior to the injury and those patients who had given informed consent. Those patients who had multiple injuries and open fractures, subtrochanteric fractures, intracapsular fractures neck of femur, pathological fractures and patients who were non-ambulatory prior to their injury were excluded. Details were taken about the duration of fracture and diagnosis as per according to their hospital guidelines. The procedure was performed by a consultant and patients were followed on regular basis as per hospital protocol. A day before follow up, telephonic calls were made to the participants for a soft reminder by the researcher. Functional outcomes were measured both during pain by using the visual analog scale and range of motion on goniometer between 80 and 100 degrees. Satisfactory outcomes were measured after three months. Satisfactory functional and radiological outcomes were considered as an acceptable outcome. Post-operative antibiotics were given for three days. The active range of motion exercises and non-weight bearing mobilization was started on the first postoperative day. Anteroposterior and lateral plain radiographs were obtained at each visit to look for the fracture union, tip apex distance, cut-out or lateral migration of helical blade. Data were entered, cleaned, coded and analyzed by IBM SPSS Statistics for Windows, Version 21.0. (IBM Corp., Armonk, NY, US). Mean ± standard deviation (SD) was calculated for the quantitative variable. Categorical variables were expressed in terms of frequency and percentage. Effect modifiers and confounding factors associated with acceptable outcome were explored by stratified analysis and chi-square test was applied to see the effect of these on functional and radiological outcomes. Two-tailed p-values were calculated and statistical significance criteria were *p*-value ≤ 0.05.

## Results

A total of 106 patients with the radiological diagnosis of unstable intertrochanteric fracture post DHS fixation were included in this study. Out of which, 69.8% (74) were male and the mean age was 66.61 ± 7.79 years over the range of 50 to 80 years. Patients with type II diabetes were 22.6% (24) with a mean duration of 4.3 ± 8.37 years. At the end of three months, no pain was reported in 82.1% (87) patients and 85.8% (91) patients had normal function. The satisfactory radiological outcome was observed in 86.8% (92) patients. Overall, acceptable outcomes were observed in 81.1% (86) patients. Baseline demographic characteristics and functional and radiologic outcomes of the study sample are presented in Table 1.

**Table 1:** Baseline demographic characteristics and functional and radiologic outcomes **Calculated based on 24 diabetic patients

Characteristics	Frequency (%) or Mean ± SD
Total patients	N = 106
Age (years)	66.61 ± 7.79
Weight (kg)	58.25 ± 8.48
Height (m)	1.76 ± 0.48
BMI (kg/m^2^)	27.58 ± 4.98
Male	74 [69.8%]
Type II diabetes	24 [22.6%]
**Duration of type II diabetes (years)	4.3 ± 8.37
Functional outcome	
No pain	87 [82.1%]
Normal function	91 [85.8%]
Satisfactory radiologic outcome	
Callus formation	92 [86.8%]
Overall acceptable outcome	86 [81.1%]

Functional and radiological outcomes of unstable intertrochanteric fracture post DHS fixation carried out after three months was statistically insignificant by gender and body mass index (BMI). However, patients with type II diabetes had relatively poor outcomes after three months as compared to non-diabetic patients with the overall acceptable outcome of 62.5% (15/24) vs. 86.6% (71/82), *p*-value = 0.008, for diabetic and non-diabetic patients respectively. Similarly, patients above 65 years of age had relatively poor outcomes after three months as compared to patients of age 50 to 65 years with the overall acceptable outcome of 72.2% (39/54) vs. 90.4% (47/52), *p*-value = 0.017, respectively. Functional and radiological outcomes after three months by baseline characteristics are presented in Table 2.

**Table 2:** Functional and radiologic outcomes after three months by baseline characteristics *Significant at 5%, **p-values are based on chi-square test

Characteristics	N	No Pain	Normal Function	Callus Formation	Acceptable Outcome
Gender					
Female	32	27 [84.4%]	26 [81.3%]	27 [84.4%]	26 [81.3%]
Male	74	60 [81.1%]	65 [87.8%]	65 [87.8%]	60 [81.1%]
**p-value	-	0.685	0.372	0.629	0.984
Type II Diabetes					
Non-diabetic	82	71 [86.6%]	76 [92.7%]	76 [92.7%]	71 [86.6%]
Diabetic	24	16 [66.7%]	15 [62.5%]	16 [66.7%]	15 [62.5%]
**p-value	-	0.025*	<0.001*	0.001*	0.008*
Age					
50 to 65 years	52	47 [90.4%]	49 [94.2%]	51 [98.1%]	47 [90.4%]
> 65 years	54	40 [74.1%]	42 [77.8%]	41 [75.9%]	39 [72.2%]
**p-value	-	0.029*	0.015*	0.001*	0.017*
Body mass index					
≤ 27 kg/m^2^	82	66 [80.5%]	70 [85.4%]	70 [85.4%]	66 [80.5%]
> 27 kg/m^2^	24	21 [87.5%]	21 [87.5%]	22 [91.7%]	20 [83.3%]
**p-value	-	0.431	0.792	0.423	0.754

## Discussion

Despite the advancements in the clinical care of the patients, management of unstable intertrochanteric fractures is a clinical challenge for orthopedic surgeons, e.g, intertrochanteric fractures are commonly combined with subtrochanteric fracture types. Their treatment is still a challenge because of the high degree of instability and it is associated with an increased rate of mortality and morbidity, especially in elderly osteoporotic patients. An increasing trend has been reported due to intertrochanteric femoral fractures and nearly half of the hip fractures are intertrochanteric fractures in elderly patients [15]. Due to a lack of strength and coordination, these fractures counter the undue stress in elderly patients while ambulating with support and crutches [16]. High rates of mortality and morbidity can be associated with prolonged immobilization and other adverse medical conditions in these patients [17].

An early restoration of patients pre-fracture activity and lifestyle is the primary goal, especially in elderly patients. However, various factors influence the functional outcomes in these patients including adequate internal fixation, minimal blood loss, minimal anesthesia time, early mobilization, and general health of the patient [16,18]. Preservation of ambulatory function is a vital part of the treatment of these fractures in patients with intertrochanteric fracture. It vastly depends on the quality of fracture stabilization, associated skeletal injuries, post-operative early ambulation, and perioperative complications [18]. Over the years, a great number of efforts have been made in the improvement of biomechanics design and implants for fixation of these fractures [18].

Dynamic hip screw (DHS) is a recommended implant designed for the fixation of unstable intertrochanteric fractures [13,19]. However, a number of complications have been reported associated with DHS fixation. Despite complications, it remains the most reliable and successful treatment option for unstable intertrochanteric fracture [20]. Although there is an increasing trend towards the use of intra-medullary devices evidence suggests that they fail to deliver better outcomes compared to DHS especially in A1 and A2 fractures [21-22].

In our study, acceptable functional and radiological outcomes of DHS fixation of unstable intertrochanteric fracture at three months were observed in 81.1% patients. Outcomes were independent of gender and body mass index of patients; nonetheless, relatively poor functional and radiological outcomes were observed in diabetic and elderly patients (>65). Our findings are comparable to past studies such as Mardani-Kivi et al. [23]. According to this study, DHS is a more reliable and successful treatment option in intertrochanteric fracture as compared to locking compression plate (LCP). Incidences of device failure and limb shortening were comparatively lesser in DHS groups after six months of follow-up. Based on the Harris Hip Score, outcomes were classified as excellent in 31.7%, good in 63.3%, and fair in 5.0% of the patients [23]. Similarly, Shetty et al. also reported good functional and radiological outcomes of DHS fixation of unstable intertrochanteric fractures with excellent to a good outcome in 59.4% as per the Harris Hip Score [24]. Another study by Barwar et al. reported excellent outcomes at one year in 45.8% and radiologically united fracture after the third month was found in 66.7% of the patients [25]. 

In contradiction to the intuition of increased risk of intertrochanteric fracture and complications after DHS fixation, we observed no statistically significant differences in functional outcomes of DHS after three months of fixation between patients with ≤27 kg/m^2^ and >27 kg/m^2^. Similarly, a systematic review of fifteen prospective cohort studies found a protective effect against hip fracture [26]. DHS results in acceptable outcomes in the majority of the patients and it is a suitable treatment option. However, further improvements in devices and techniques are warranted to reduce complication rates.

## Conclusions

The treatment of unstable intertrochanteric fracture with DHS fixation results in better outcomes. In our study, we observed acceptable outcomes in the vast majority, 81.1%, of patients after three months of DHS fixation of the unstable intertrochanteric fracture.
